# Mechanistic Studies of the Anti-Ulcerogenic Activity and Acute Toxicity Evaluation of Dichlorido-Copper(II)-4-(2-5-Bromo-benzylideneamino)ethyl) Piperazin-1-ium Phenolate Complex against Ethanol-Induced Gastric Injury in Rats

**DOI:** 10.3390/molecules16108654

**Published:** 2011-10-14

**Authors:** Muhammad Saleh Salga, Hapipah Mohd Ali, Mahmood Ameen Abdullah, Siddig Ibrahim Abdelwahab, Pouya Davish Hussain, A. Hamid A. Hadi

**Affiliations:** 1 Department of Chemistry, University of Malaya, 50603 Kuala Lumpur, Malaysia; 2 Department of Molecular Medicine, University of Malaya, 50603 Kuala Lumpur, Malaysia; 3 Department of Pharmacy, University of Malaya, 50603 Kuala Lumpur, Malaysia

**Keywords:** Schiff bases, acute toxicity, anti-ulcer activity, mechanism, cytokines

## Abstract

The compound dichlorido-copper(II)-4-(2-5-bromobenzylideneamino)ethyl) piperazin-1-ium phenolate (CuLBS) was synthesized, characterized and screened for acute toxicity and protective activity against ethanol-induced gastric mucosal injury in rats. Gross microscopic lesions, biochemical and immunological parameters and histochemcial staining of glycogen storage were taken into consideration. Oral administration of CuLBS (30 and 60 mg/Kg) for two weeks dose-dependently flattened gastric mucosa, significantly increased gastric mucus and total acidity, compared with control group (*P < 0.01*). Serum levels of liver enzymes aspartate (AST) and alanine transaminases (ALT), pro-inflammatory (IL-6 and TNF-α) and anti-inflammatory (IL-10) cytokines in the rats exposed to ethanol induced ulceration have been altered. Administration of CuLBS showed considerable (*P < 0.05*) protection against ulceration by modulating the acute alterations of cytokines AST, ALT and stomach glycogen. Interestingly, CuLBS did not interfere with the natural release of nitric oxide. CuLBS alone (60 mg/Kg) did not exhibit any ulcerogenic effect as assessed using Adami’s scoring scale. An acute toxicity study showed that rats treated with CuLBS (1,000 and 2,000 mg/Kg) manifested no abnormal signs. These findings therefore, suggested that the gastroprotective activity of CuLBS might contribute in modulating the inflammatory cytokine-mediated oxidative damage to gastric mucosa.

## 1. Introduction

The inflammation caused by ethanol-induced gastric injuries has now become a standard measure for testing the therapeutic potencies of many synthetic compounds. Studies on compounds with anti-ulceragenic activities, both synthetic and those isolated from plant extracts, is now attracting the interest of many researchers [1] The use of ethanol as an ulcer model on experimental rats was observed to caused an intense infiltration of submucosa, affect the production of nitric oxide synthase, enhance the formation of reactive oxygen species (ROS), deplete mucus membrane, gastric pH and blood flow and subsequently damage of gastric mucosa ensued. The pathogenesis caused by this ulcer model also involved inhibition of the enzyme cyclooxygenase, which in turn suppressed the output of endogenous prostaglandins leading to changes in gastric motility [2]. Peptic ulcers have now become a global problem and one of the most common chronic illnesses among youths [3]. The therapeutic potentials of many compounds can be evaluated according to how they act to enhance gastric protection from the attack of ulcer models. Compounds containing a piperazine moiety have showed therapeutic potency in many biological studies [4,5] and the efficacy of such compounds was observed to increase after complexation [6]. Moreover, piperazine derivatives afford biologically active compounds against enzyme and receptor targets, for example a potent HIV protease inhibitor indinavir that has been approved for use in man or clozapine, an antipsychotic agent that blocks dopamine and potent MC4-receptor, to mention a few examples where a piperazine core has been used as a scaffold to produce biologically active compounds. This makes the piperazine core a privileged motif for drug design [7]. Meanwhile, Schiff bases are considered to be an important class of organic compounds in medicinal and pharmaceutical chemistry [8,9] and the exploration of novel Schiff base derivatives with chemotherapeutic potential is attracting the attention of many chemists [10]. Schiff bases containing piperazine heterocyclic ring (moieties) were reported to possess a broad spectrum of biological activities such as antiviral [11], anticancer [12], anticonvulsant, acetylcholinesterase inhibition [13] anti-PAF [14] anti-HIV [15], *etc.* Generally, Schiff bases show fascinating chelating behavior in coordinating with metal ions which makes them important radiopharmaceuticals for cancer treatment, in agrochemicals and as models for biological systems [16,17].

Copper is an essential element that serves as a cofactor for metalloenzymes that bind plasma to form cerloplasmin found in the liver [18]. Copper is involved in many biological systems [19,20,21] and this has attracted the attention of many researchers to determine its mechanisms of absorption [22,23] distribution [24] metabolism, and excretion [25]. The diseases cause by copper deficiency or excess were also investigated [26,27]. This study therefore, aimed to synthesize, characterize and evaluate the copper complex derived from the Schiff base bromosalicylaldimino-ethylpiperazine for acute toxicity and protective activity against ethanol-induced gastric injuries.

## 2. Results and Discussion

**Figure 1:**
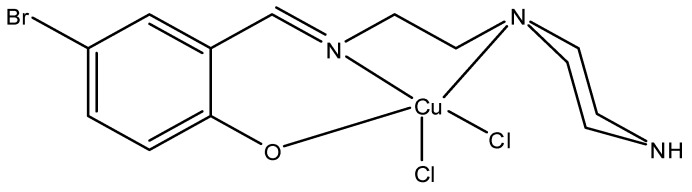
Molecular structures of CuLBS complex.

The reaction of 5-bromosalicylaldehyde with 2-(piperazin-1-yl)ethanamine produces the Schiff base (*E*)-4-bromo-2-((2-(piperazin-1-yl)ethylimino)methyl)phenol, while treatment of this Schiff base with copper(II) chloride resulted in the formation of compound CuLBS. The IR spectra of the Schiff base showed absorption at 1,638 cm^−1^ which can be afforded to azomethine. This absorption shifted to 1,633 cm^−1^ in the spectra of CuLBS due to complexation (Figure 1). This is confirmed at 640 nm, ascribed to metal-imine (M–N) bond formation. The UV-visible spectra also indicate absorption at 636 nm that can be attributed to d-d electronic transition in the compound CuLBS. This absorption was absent in the spectra of the Schiff base. However, the ^1^H-NMR spectra of the compound CuLBS was not clear possibly due to paramagnetic nature of the copper ion.

**Figure 2:**
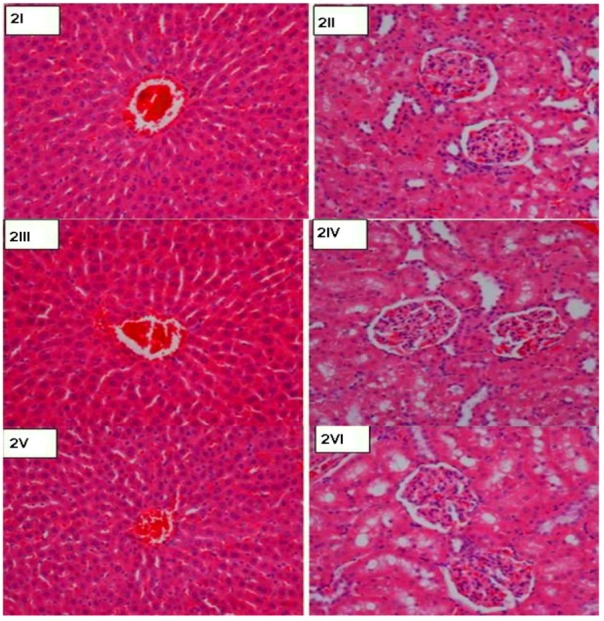
Histological sections of liver and kidney in acute toxicity test. (**2I** and **2II**) Rats treated with 5 mL/Kg vehicle (CMC); (**2III** and **2IV**) Rats treated with 1 g/Kg (5 mL/Kg) CuLBS; (**2V** and **2VI**) Rats treated with 2 g/Kg (5 mL/Kg) CuLBS. There is no significant differences in morphological structures of liver and kidney between treated and control groups (H & E stain 20×).

**Figure 3:**
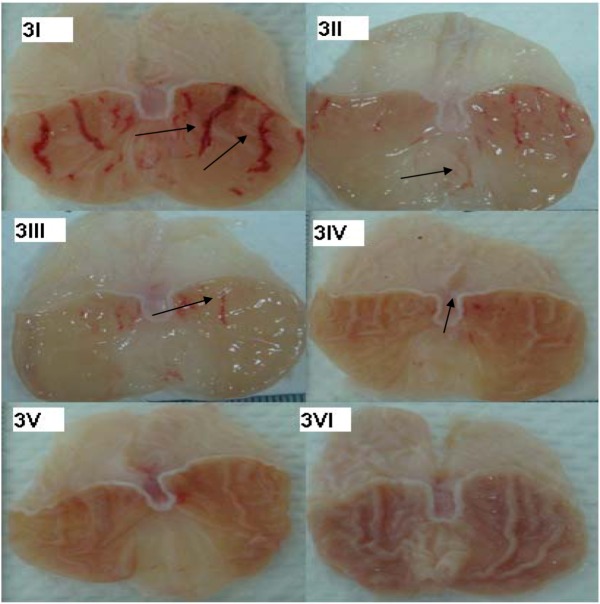
Gross appearance of the gastric injuries in rats. (**3I**) Rats pre-treated with 5 mL/Kg distilled water (ulcer control). Severe injuries are seen in the gastric mucosa (arrow). Absolute ethanol produced extensive visible hemorrhagic necrosis of gastric mucosa; (**3II**) Rats pre-treated with omeprazole (20 mg/Kg). Injuries to the gastric mucosa are very milder (arrow) compared to the injuries seen in the ulcer control rats; (**3III**) Rat pre-treated with CuLBS (30 mg/Kg) and then followed with ethanol (CuLBS-Et, 30 mg/Kg). Mild to moderate injuries are seen in the gastric mucosa (arrow). The compound reduces the formation of gastric lesions induced by absolute ethanol; (**3IV**) Rats pre-treated with CuLBS (60 mg/Kg) and then followed with ethanol (CuLBS-Et, 60 mg/Kg) Very mild injuries are seen in the gastric mucosa (arrow); (**3V**) Rats pre-treated with CuLBS alone (60 mg/Kg), no injuries to the gastric mucosa are seen (**3VI**). The normal rats, no injuries to the gastric mucosa are seen.

The oral administration of the compound CuLBS for 14 days resulted in no significant changes in the weight of the rats utilized in this study (Table 1). The results obtained from histological evaluations of liver and kidney showed no significant differences between the control and the rats pre-treated with CuLBS (Figure 2I–VI). In addition, the biochemical parameters for renal and liver functions of the normal rats and the rats pre-treated with CuLBS were in close agreement (Table 2 and Table 3). The gastroprotective efficacy of the compound was further evaluated against ethanol-induced gastric ulcerations in rats. Pre-treatment with 30 mg/Kg and 60 mg/Kg were found to inhibit gastric mucosal injury caused by ethanol (Figure 3III and Figure 3IV). This inhibitory effect was highest in the 60 mg/Kg group, which indicates that the compound protects the gastric mucosa dose-dependently. Similarly, 30 mg/Kg of CuLBS significantly inhibited ethanol-induced gastric lesions compared to the control.

**Table 1:** Effects of CuLBS on body and organ weights.

Groups	I	II	III	IV	V	VI
Body wt	190 ± 11.0	200 ±9.00	205 ± 10.0	215 ± 10.0	195 ± 10.0	193 ± 8.00
Liver wt	2.89 ± 0.20	2.96 ± 0.18	3.21 ± 0.21	3.39 ± 0.19	2.92 ± 0.10	3.11 ± 0.20
Kidney wt	1.34 ±0.03	1.29 ± 0.02	1.36 ± 0.05	1.39 ± 0.03	1.32 ± 0.02	1.35 ± 0.04

**Figure 4:**
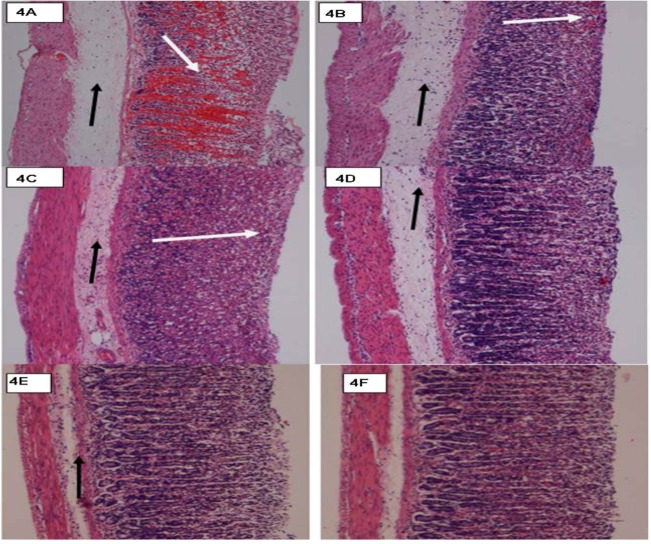
Histological view of the ethanol-induced gastric mucosal damage in rats. (**4I**) Rats pre-treated with 5 mL/Kg of distilled water (ulcer control). There is severe disruption to the surface epithelium and necrotic lesions penetrate deeply into mucosa (white arrow) and extensive edema of submucosa layer and leucocyte infiltration are present (black arrow); (**4II**) Rats pre-treated with omeprazole (20 mg/Kg). Moderate disruption of the surface epithelium mucosa is present (white arrow) but deep mucosal damage is absent. Submucosal edema and leucocytes infiltration (black arrow); (**4III**) Rat pre-treated with CuLBS (30 mg/Kg) and then followed with ethanol (CuLBS-Et, 30 mg/Kg). Mild disruption of surface epithelium is present but deep mucosal damage is absent. Submucosal edema and leucocytes infiltration (black arrow); (**4IV**) Rat pre-treated with CuLBS (60 mg/Kg) and then followed with ethanol (CuLBS-Et, 60 mg/Kg) Very mild disruption of surface epithelium is present but deep mucosal damage is absent. Submucosal edema and leucocytes infiltration (black arrow); (**4V**) Rats pre-treated with CuLBS-alone (60 mg/Kg).There are no disruption to the surface epithelium with no edema and no leucocytes infiltration of the submucosal layer; (**4VI**) The normal rats rats pre-treated with CMC alone. There is no disruption to the surface epithelium with no edema and no leucocytes infiltration of the submucosal layer (H & E stain; 10×).

**Figure 5:**
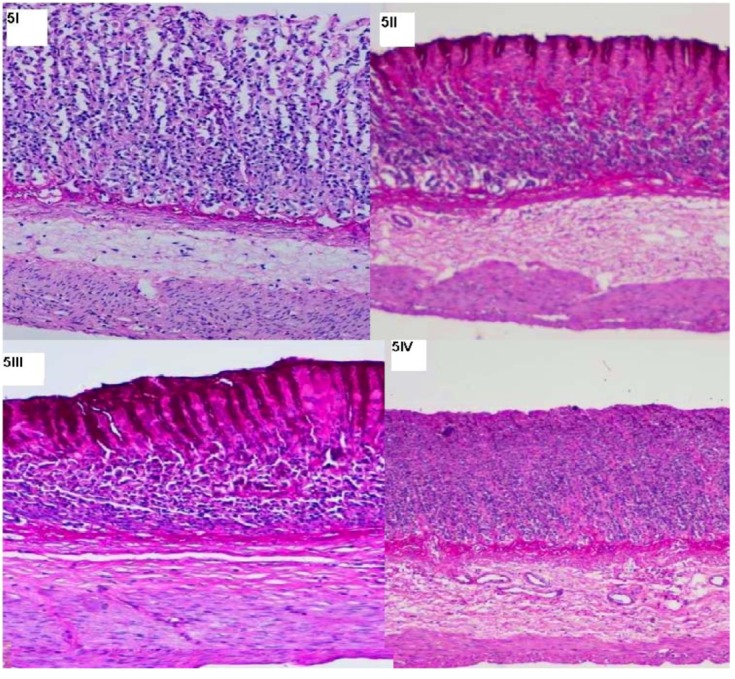
Effect of CuLBS on gastric tissue glycogen-PAS staining. Stomach subjected to ethanol-induced ulcer after treatment with CMC (**5I**); CuLBS (30 mg/Kg) (**5II**), CuLBS (60 mg/Kg) (**5III**) and the normal rat stomach (**5IV**).

The gastric mucus, which consists of a viscous, elastic, adherent and transparent gel formed by water and glycogens covering the entire gastrointestinal mucosa plays an important role in protecting it. The protective activity of the mucus barrier relies on that gel structure and the thickness of the layer covering the mucosal surface [28]. This study found that the compound CuLBS enhanced the production of gastric adherent mucus (Figure 4III and Figure 4IV), increased mucus secretion and decrease gastric acidity at both doses. This enables the mucus layer to protect the newly formed cells against damage caused by ethanol (Figure 5III and Figure 5IV).

**Table 2:** Renal function test of rats in acute toxicity study of CuLBS compound.

Animals	Na^+^ (mmol/L)	K^+^ (mmol/L)	Cl^−^ (mmol/L)	CO_2_ (mmol/L)	AG (mmol/L)	Urea (mmol/L)	Crt (µmol/L)
Control	132 ± 3.1	6.31 ± 1.3	120 ± 1.4	26 ± 1.5	20.8 ± 1.5	6.2 ± 1.6	45.8 ± 2.4
LD (1 g/Kg)	131 ± 2.4	6.42 ± 1.2	119 ± 1.7	25 ± 1.5	21.8 ± 1.6	6.5 ± 1.9	43.9 ± 2.6
HD (2 g/Kg)	134 ± 2.2	6.63 ± 1.4	122 ± 1.3	28 ± 1.2	21.6 ± 1.4	6.4 ± 1.3	46.3 ± 2.2

Values expressed as mean ± S.E.M. There are no significant differences between groups. Significant value at p < 0.05.

**Table 3:** Liver function test of rats in acute toxicity study of CuLBS compound.

Animals	T. Prot. (g/L)	Albumin (g/L)	Globulin (g/L)	TB (µmol/L)	CB (µmol/L)	AP (IU/L)	ALT (IU/L)	AST (IU/L)
Control	78 ± 1.3	46.2 ± 1.8	39.6 ± 1.3	9.5 ± 1.8	1	128 ± 1.91	62 ± 1.3	249 ± 3.2
LD (1 g/kg)	77 ± 1.6	47.2 ± 1.3	37.8 ± 1.4	9.6 ± 1.6	1	127 ± 1.62	65 ± 1.4	248 ± 3.5
HD (2 g/Kg)	79 ± 1.8	48.5 ± 1.4	38.7 ± 1.6	9.8 ± 1.2	1	129 ± 1.67	64 ± 1.6	251 ± 3.1

Values expressed as mean ± S.E.M. There are no significant differences between groups. Significant value at p < 0.05 T. Prot; total protein, TB: Total bilirubin; CB: Conjugated bilirubin; AP: Alkaline phosphatase; ALT: Alanine aminotransferase; AST: Aspartate aminotransferase; AG: anion gap; Crt: creatinine.

The ethanol model is widely used to assess the protective and healing activity of many drugs in ulcer studies [29] due to its ability to reduce endogenous NO level and blood flow in gastric mucosa, which leads to a serious hemorrhagic necrosis and consequently depletes gastric mucus constituents [30] resulting in an increased flow of Na^+^ and K^+^, elevated pepsin secretion, loss of H^+^ ions and histamine into the lumen [31]. In our effort to investigate the mechanism involved in the action of this compound, we evaluated the level of liver enzymes AST, ALT, the energy storage creatinine, the high density lipo-protein and the leukocyte count between the ethanol group and the groups pre-treated with CuLBS. The results showed that rats induced with ethanol ulceration had increased level of liver enzymes AST and ALT compared to normal and the rats that received CuLBS, this rise was more noticeable with AST which can be ascribed to alcoholic hepatitis due to ethanol. Pre-treatment with CuLBS dose-dependently reduces this liver toxicity caused by ethanol. The creatinine level, high density lipoprotein, leukocyte count and c-reactive enzymes were also altered in the acute ulcerated rats compared to the normal and rats pre-treated with CuLBS (Table 4). However, the rats pretreated with CuLBS showed a decreased in such parameters. This can presumably be due to high activity of the compound to protect the gastric from ethanol induced injuries. Moreover, pre-treatment with CuLBS to ulcer induced rats has led to the flattening of mucosal folds, increased pH and gastric mucus content (Table 5) ensuring the protective activity of this novel copper complex.

**Table 4:** Biochemical parameters in gastroprotectivity study of CuLBS.

Animals	Treatments	ALT (IU/L)	AST (IU/L)	C-reactive	HDL	Creatinine	WBC
I	CMC (ulcer control)	94.23 ± 8.2	296.2 ± 9.2	0.18 ± 0.04	0.09 ± 0.4	21.2 ± 1.9	4.16 ± 2.1
II	Omeprazole (20 mg/Kg)	65.22 ± 4.2	189.4 ± 7.6	0.63 ± 0.10	1.22 ± 0.5	47.9 ± 9.2	9.23 ± 3.2
III	CuLBS-Et-(30 mg/Kg)	67.31 ± 3.4	193.3 ± 8.4	0.65 ± 0.09	1.24 ± 0.3	48.6 ± 1.5	9.31 ± 2.7
IV	CuLBS-Et-(60 mg/Kg)	68.42 ± 5.2	195.7 ± 7.2	0.67 ± 0.04	1.26 ± 0.3	51.3 ± 2.3	10.21 ±3.2
V	CuLBS-alone-0 mg/Kg	59.61 ± 4.6	196.5 ± 6.4	0.55 ± 0.06	1.20 ± 0.4	45.2 ± 9.6	9.11 ± 1.3
VI	Normal	64.28 ± 4.7	194.9 ± 7.1	0.69 ± 0.07	1.25 ± 0.6	52.6 ± 7.3	10.61 ± 2.3

**Table 5:** Observed ulcer area and %Inhibition.

Animal	Pretreatment (5 mL/Kg)	Gastric pH	Mucus wt (g)	Ulcer area (mm)	%Inhibition
I	CMC (ulcer control)	1.43 ± 0.7	0.95 ± 1.3	962.19 ± 12.62	-
II	Omeprazole (20 mg/Kg)	4.92 ± 0.6	3.65 ± 1.2	189.24 ± 9.6	80.30
III	CuLBS-Et- (30 mg/Kg)	5.36 ± 1.2	4.47 ± 2.3	125.62 ± 5.3	86.94
IV	CuLBS-Et- (60 mg/Kg)	6.98 ± 1.4	5.31 ± 2.8	104.45 ± 4.6	89.14
V	CuLBS-alone- 60 mg/Kg	5.14 ± 0.9	3.82 ± 1.6	0	100
VI	Normal	3.28 ± 1.9	3.28 ± 1.8	0	100

Ulcer area expressed as (mean + S.E.M).

Interleukins play an important role in the regulation of the mucosal defense barrier. Levels of pro-inflammatory cytokines such as IL-6 and TNF-α in the gastric mucosa were significantly increased while the anti-inflammatory IL-10 was significantly decreased in gastric injury models (Table 6). However, in ethanol induced rats, the anti-inflammatory marker IL-10 decreased, whereby the CuLBS treated groups inhibited the depletion of this cytokine, which proves the role played by CuLBS in gastric protection. To describe the effect of CuLBS on the gastric inflammation, the level of inflammatory mediator (NO) and lipid peroxidation were evaluated. Ethanol induction notably decreased the level of NO, but pre-treatment with CuLBS enhances the production of NO and inhibit lipid peroxidation. It was reported that nitric oxide (NO) synthesis protect the gastric mucosa against damage induced by various ulcer models and that the serum and local NO levels are reduced in gastric injury models induced by ethanol and indomethacin [32]. This indicates that the decrease in local NO content might be a key factor in facilitating gastric mucosal injury [33].

**Table 6:** Effects of CuLBS on MDA, NO, TNF-α, IL-6 and IL-10 in ulcerated in rats.

Animals	MDA (µmol/g Tissue)	NO (µmol)	TNF-α (pg/mg)	IL-6 (pg/mg)	IL-10 (pg/mg)
I	25.32 ± 1.27 ^a^	3.32 ± 0.03	361 ± 3.8	67 ± 2.1	72 ± 6.2
II	13.65 ± 1.49 ^b^	7.85 ± 0.04	94.0 ± 2.8	10.4 ± 1.5	264 ± 4.5
III	12.21 ± 1.32 ^c^	9.42 ± 0.09	45.0 ± 4.5	6.20 ± 1.8	289 ± 3.3
IV	10.25 ± 0.85 ^c^	9.92 ± 0.85	21.4 ± 0.4	4.50 ± 1.6	342 ± 2.1
V	14.16 ± 1.23 ^c^	6.83 ± 1.20	0	0	258 ± 5.2
VI	12.51 ± 1.12 ^c^	8.93 ± 1.13	0	0	239 ± 6.5

Group I: CMC + EtOH; GroupII: animals that received omeprazole + ethanol; Group III: animals that received CuLBS (30 mg/Kg + EtOH); Group IV: animals that received CuLBS (60 mg/Kg + EtOH), Group V: animals that received CuLBS (60 mg/Kg) alone; Group VI: Normal animals.

## 3. Experimental

### 3.1. General

Elemental analysis was conducted on Costech Elemental Combustion System CNHS-O elemental analyzer, NMR was performed on ECA-400 higher performance FTNMR Spectrophotometer, IR spectra were recorded on Perkin-Elmer FTIR Spectrophotometer, UV-visible spectroscopy was carried out on Perkin-Elmer-1650-UV-visible spectrophotometer. 5-bromosalicylaldehyde, 2-(piperazin-1-yl)ethanamine, copper(II) chloride salt, solvents and all other chemicals were purchased from Merk-Aldrich, Kuala Lumpur, Malaysia.

### 3.2. Synthesis and Isolation of CuLBS

To a stirred solution of 2-(piperazin-1-yl)ethanamine (1.29 g, 10 mmol) in ethanol (25 mL) an equimolar stirred solution of 5-bromosalicylaldehyde (2.0 g, 10 mmol) in ethanol (25 mL) was added dropwise at room temperature and then refluxed for 3 h at 75–85 °C. An orange solution was obtained, which after removing the solvent gave a red oil. After stabding for 3 days the red oil forms a hygroscopic solid which is dissolved in methanol (50 mL) by heating to a temperature of 60–65 °C followed by addition of a few drops of sodium hydroxide (1 M). A few drops of diethyl ether were added to the solution while it is hot, and a reddish-yellow solid appeared, which is filtered off immediately and dried under vacuum. The solid was recrystallized from methanol and collected after concentrating and cooling, washed with ethanol-water mixture (20:80) and dried under vacuum. The compound was characterized by IR, NMR and UV-visible spectroscopy. The pH and pKa of the compound was also determined. Yield: (0.24 g, 76.9%). IR (KBr disc cm^−1^): υ(NH–/OH), 3421sb; υ(C–H), 2736s; υ(C=N), 1638s, υ(C–C), 1458s; υ(C–N), 1176s; υ(Br–Ar), 688s. ^1^H-NMR (DMSO-*d*6), δ, ppm: 2.0 (s, 1H, NH), 2.48 (t, 2H, 2CH_2_), 6.78–7.02 (d, 1H, ArH), 8.54 (s, 1H, imine), 11.26 (s, 1H, phenolic) ^13^C-NMR (DMSO-*d*6), δ, ppm: 18.96 (CH_3_), 39.84–40.47 (DMSO-*d*6 + CH_2_), 54.51-59.06 (CH_2_), ArC: [108.95 (CH), 120.00 (CH), 133.90 (CH), 135.36 (CH), 161.77 (CH), 165.65 (CH)]; *m/z*: 310.09, 311.02, 314.07. pH, 9.13; pKa, 2.73. UV-Vis (DMSO), λ_max_ (ε, Mol^−1^cm^−1^): 279 nm (3091.79, π-π*); 303 nm (3152.21, CT).

To a measured quantity of the synthesized Schiff base (0.312 g, 1 mmol) dissolved in methanol (25 mL) was added a weighed quantity of copper(II) chloride (0.17 g, 1 mmol) also in methanol (25 mL) at room temperature with stirring, followed with a few drops of potassium hydroxide (1 M). The solution immediately forms a green precipitate that was filtered off, washed with ethanol-water mixture (80:20) and dried in a vacuum desiccator, yield (0.34 g (76.4%)). Anal. Calc. for C_13_H_17_BrCl_2_CuN_3_O: C, 35.04; H, 3.84; N, 9.43. Found; C, 30.76; H, 3.24; N, 8.46. IR (KBr disc cm^−1^): υ(NH–/OH), 3434s; υ(C–H), 2737s; υ(C=N), 1633s, υ(C–C), 1457s; υ(C–N), 1177s; υ(phenyl ring), 906; υ(M–O), 690s; υ(M–N), 640s. UV-Vis (DMSO), λ_max_ (ε, mol^−1^cm^−1^): 274 nm (2527.6, π-π*); 377 nm (1,535.2, LMCT); 636 nm (142.62, d-d). The proposed structure is shown in Figure 1.

### 3.3. Animals

Males Sprague Dawley rats weighing about 180 ± 50 g were obtained from the Animal House, Faculty of Medicine, University of Malaya (Kuala Lumpur, Malaysia). Rats were distributed into six groups of six rats each. The animals were fed with a standard pellet diet and tap water under controlled temperature conditions (22 ± 4 °C). Animals received human care according to approved institutional guidelines and experiments formulated by the National Academy of Sciences and published by the National Institute of Health, Malaysia.

### 3.4. Estimation of Oral Acute Toxicity

The acute toxicity of CuLBS was estimated according to the reported guidelines for testing of chemicals acute oral toxicity [34] using 2,000 mg/kg (high dose) and 1,000 mg/kg (low dose) body weight (b.w). Adult male and female Sprague Dawley rats (6–8 weeks old; 150–180 g) were obtained from the Experimental Animal House [Ethics No. PM 07/05/2008 MAA (a)(R)], Faculty of Medicine, University of Malaya. The animals were given standard food and clean water prior to dosing and then fasted 18 h before the administration of compound CuLBS. The overnight fasted animals were treated with CuLBS at doses of 1,000 and 2,000 mg/kg b.w., and the food was withheld for further 3–4 h after dosing. The animals were kept under observation for 14 days (Table 1) to examine any clinical, toxicological symptoms or mortality. The animals were sacrificed on the 15th day and all the biochemical parameters (Table 2 and Table 3) and histological data (Figure 2) were analyzed.

### 3.5. Ulcerogenic Study

Group I and VI rats were given food and water for 14 days [35], Group II rats were treated with omeprazole dissolved in carboxymethylcellulose (CMC) and Group III and Group IV rats were orally administered CuLBS dissolved in carboxymethylcellulose (CMC) at 30 and 60 mg/Kg b.w., respectively, for 14 days. Rats in Group V received CuLBS alone for 14 days. The animals were fasted for 24 h on day 14. On day 15, group II, received omeprazole, Group III, IV and V were given CuLBS while Group I and VI received vehicle. One hour after this treatment, animals in Group I, II, III, IV and V received orally 95% ethanol at the dose of 5 mL/Kg. The animals were sacrificed 30 min latter by cervical decapitation under anesthetized xylazin and ketamine. Serum was obtained for biochemical analyses.

### 3.6. Serum Biochemical Assays

Blood samples were analyzed at University Malaya Medical Centre to evaluate changes in biomarkers.

### 3.7. Measurement of Gastric Juice Acidity

The stomachs of animals sacrificed were excised. The gastric contents was collected, centrifuged at 4,000 rpm and the supernatant used for the determination of hydrogen ion concentration by titration with 0.1 N NaOH solution using digital pH meter. The acid content was expressed as mEq/L [36].

### 3.8. Histopathological Studies

The histological alterations observed in the gastric of ulcerated rats were assessed by fixing the gastric walls from each rat in 10% buffered formalin and processed in a Paraffin Tissue Processing Machine. Sections of the gastric were made at a thickness of 5 µM and stained with hematoxylin and eosin for histological evaluation.

### 3.9. Gastroprotective Assessments

Elongated bands of hemorrhagic lesions parallel to the long axis of the stomach were observed in the gastric of animals that received ethanol. The length (mm) and width (mm) of the ulcer on the gastric mucosa were measured using planimeter [(10 × 10 mm^2^ = ulcer area) under dissecting microscope (1.8×)]. The area of each ulcer lesion was measured by counting the number of small squares, 2 mm × 2 mm, covering the length and width of each ulcer band. The sum of the areas of all lesions for each stomach was applied in the calculation of the ulcer area (UA) wherein the sum of small squares × 4 × 1.8 = UA mm^2^. The inhibition percentage (I%) was calculated as described in [37] with slight modifications by the following formula:

Inhibition percentage (I%) = [(UAcontrol − UAtreated) / UAcontrol] × 100%.

### 3.10. Gastric Mucus Evaluations

The gastric content was collected and centrifuged at 4,000 rpm for 10 min. The supernatant was removed and the mucus weighed using a precision electronic balance.

### 3.11. PAS Staining

To examine the extent of protection provided by CuLBS against ethanol-induced gastric lesions, gastric tissues were stained histochemically to evaluate the mucus content. Formalin fixed paraffin and embedded gastric tissues were sectioned, dewaxed and stained using a commercial PAS staining system kit (Sigma Aldrich, Malaysia) according to the manufacturer’s instructions [38].

### 3.12. Estimation of the Role of Total Nitric Oxide

The concentration of nitric oxide in the gastric homogenate was evaluated as total nitrate/nitrite levels using the Griess reagent [39]. The stomach homogenates in 50 mM potassium phosphate buffer (pH 7.8) were centrifuged at 4,000 rpm for 30 min at 4 °C. Fifty μL of the Griess reagent (0.1% *N*-(1-naphthyl) ethylenediamidedihydrochloride, 1% sulfanilamide in 5% phosphoric acid) was added to supernatant (50 μL) and mixed, then after 10 min the absorbance was measured at 540 nm. The standard curves were obtained by using sodium nitrite. Results were expressed as micromoles nitrate/nitrite per gram of protein.

### 3.13. Estimation of Membrane Lipids Peroxidation

To evaluate the level of lipid peroxidation in the mucus membrane, the level of malondialdehyde was estimated using the thiobarbituric acid-reactive substances (as indicators of lipid peroxidation) assay according to the reported procedure [40] with some modifications. The stomach homogenates were mixed with a solution (0.125 mL) containing 26 mM thiobarbituric acid, 0.26 M HCl, 15% trichloric acid and 0.02% butylatedhydroxytoluene. The mixtures were heated at 96 °C for 15 min and centrifuge at 4,000 rpm for 10 min. The supernatant was transferred to 96-well plate and the absorption was measured at 532 nm using tetramethoxypropane as standard.

### 3.14. Estimation of IL-6, IL-10 and TNF-α

To evaluate the extent at which the compound CuLBS reduce the inflammatory effects of ethanol- induced lesions in the ulcerated rats, the level of cytokines (IL-6, IL-10 and TNF-α) in the serum were evaluated using ELISA kits; ELR-IL6, IL-10 and TNF-alpha-001 for rats according to the manufacturer’s (Ray Biotech, Norcross GA, USA) instructions. Primary antibodies were first coated on the well plate and after washing, each well was blocked to remove the non-specific binding. One hundred microliters of sample or cytokine standards were added to each well and then followed with biotin conjugated secondary antibodies. To obtain a color reaction, streptavidin-HRP and substrate solution were added. The absorbance was measured at 450 nm with anan ELISA reader (TECAN, Mannedorf, Switzerland). Standard curves were plotted on each assay plate using recombinant IL-6, IL-10 and TNF-α in serial dilution.

### 3.15. Estimation of Gastric Tolerance

The ethanol induced gastric injuries were examined under an illuminated magnifier (3×) and scored according to a modified scoring system of Adami *et al.* [41] (0: no lesions; 0.5: slight hyperaemia or ≤5 petechiae; 1: ≤5 erosions ≤5 mm in length; 1.5: ≤5 erosions ≤5 mm in length and many petechiae; 2: 6–10 erosions ≤5 mm in length; 2.5: 1–5 erosions >5 mm in length; 3: 5–10 erosions >5 mm in length; 3.5: >10 erosions >5 mm in length; 4: 1–3 erosions ≤5 mm in length and 0.5–1 mm in width; 4.5: 4–5 erosions ≤5 mm in length and 0.5–1 mm in width; 5: 1–3 erosions >5 mm in length and 0.5–1 mm in width; 6: 4 or 5 grade 5 lesions; 7: ≥6 grade 5 lesions; 8: complete lesion of the mucosa with haemorrhage).

### 3.16. Statistical Analysis

All values were reported as mean ± S.E.M. The statistical significance of differences between groups was assessed using one-way ANOVA. A value of p *< 0.05* was considered significant.

## 4. Conclusions

In conclusion, the compound CuLBS was observed to show protective activity by preserving the level of NO synthase, increasing the level of anti-inflammatory cytokine IL-10 and high density lipoprotein, inhibiting lipid peroxidation and decreasing the levels of pro-inflammatory cytokines IL-6 and TNF-α. Such outcomes can be used as a guide for further research to determine the exact mechanistic action of this novel compound.
